# Primary Small Intestinal Lymphoma Presenting as a Groin Abscess

**DOI:** 10.4103/1319-3767.45066

**Published:** 2009-01

**Authors:** Ahmed N. Assar

**Affiliations:** General Surgery, Luton and Dunstable Hospital, Lewsey Road, Luton-LU4 0DZ, Bedfordshire - United Kingdom. E-mail: mrassar@hotmail.com

Sir,

Small bowel cancers are rare; primary small intestinal lymphoma accounts for 15–20% of these tumours. Patients with small intestinal lymphoma frequently present with abdominal emergencies to the surgeon.[1]

**Figure 1:**
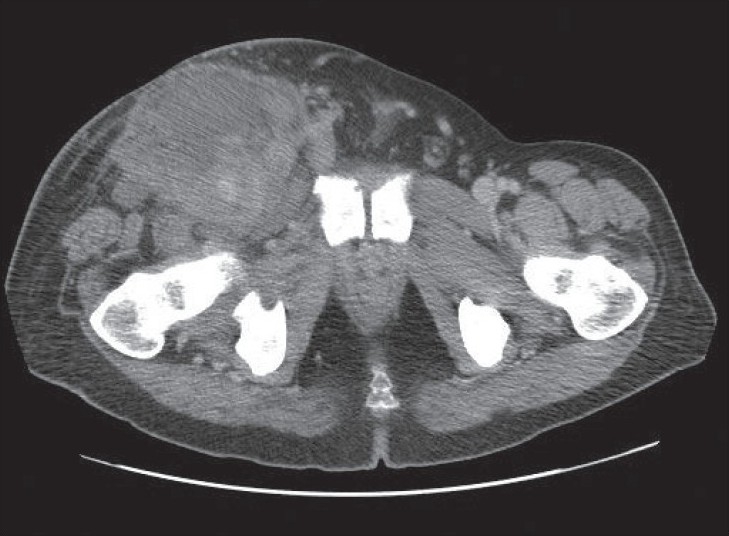
CT image showing mass extending into the right side of the pelvis

A 50-year-old man presented with a 6-month history of a right groin swelling. On further questioning, the patient reported weight loss and reduced appetite. He reported no abdominal pain and changes in bowel habits. Physical examination revealed a large right groin swelling measuring approximately 15 cm in diameter. The swelling had the features of an abscess (red, hot, tender, and fluctuant) extending downwards into the right thigh and upwards into the right lower quadrant of the abdomen. Laboratory tests showed a mild leucocytosis of 12.3 × 109/L, a haemoglobin concentration of 11.4 g/100 mL and a C-reactive protein of 311 mg/L. A computed tomography (CT) scan revealed a soft tissue mass measuring 12 cm × 12 cm × 23 cm occupying the right hemipelvis and extending into the right inguinal region [Figure 1]. There was no thoracic or abdominal lymphadenopathy.

The patient underwent incision and drainage of this abscess. There was a large amount of faeculant material filling the entire abscess cavity; this appeared to be extending upwards medial to the femoral vessels, along the femoral canal. Laparotomy through a right lower transverse incision was made. Surprisingly, there was no intraperitoneal faecal contamination. Exploration revealed a full thickness retroperitoneal perforation of the terminal ileum with fecal contamination of the retroperitoneal space of the right iliac fossa. It was evident that part of the circumference of the terminal ileum was incarcerated in the femoral ring resulting in a femoral hernia and consequent perforation. An ileocaecal resection was performed. Histopathological examination confirmed diffuse large B-cell lymphoma. Two months later, a repeat CT scan showed a reduction of the right pelvic mass to 7.5 cm × 12 cm, with no evidence of lymph node involvement elsewhere. The patient has currently fully recovered from surgery and is receiving chemotherapy.

The gastrointestinal tract is the most common site of extranodal non-Hodgkin lymphoma.[1] In 20–30% of cases, the small intestine, particularly the ileum, is affected.[2] Small intestinal lymphomas most commonly cause abdominal pain (82%).[3,4] Other presenting symptoms include changes in bowel habits (16%), a palpable abdominal mass (16%), and blood in the stool (15%).[2] In approximately 30–50% of patients, however, the initial presentation of small bowel lymphoma is an abdominal emergency,[1,5] with perforation present in up to 37% of cases.[5]

A review of literature in English revealed no cases of small intestinal lymphoma presenting as a groin abscess. What was unique about this patient was that despite having a small bowel perforation, he did not present with an abdominal emergency. As the perforation was extraperitoneal, the presentation was that of a chronic right groin swelling, which was diagnosed clinically as an abscess. The duration of the symptoms before seeking medical advice was also unusually long (6 months). This perhaps reflected the patients own beliefs and character. This is believed to be the first documented case of primary small intestinal lymphoma causing a groin abscess as a result of intestinal perforation. This case highlights the variable and somewhat unusual presentation of these tumours.
